# A Novel Heterozygous Intronic FBN1 Variant Contributes to Aberrant RNA Splicing in Marfan Syndrome

**DOI:** 10.1002/mgg3.70004

**Published:** 2024-09-02

**Authors:** Djouhayna Dougarem, Yi‐Xiao Chen, Yi‐Na Sun, He‐Feng Huang, Qiong Luo

**Affiliations:** ^1^ School of Medicine Zhejiang University Hangzhou China; ^2^ Key Laboratory of Reproductive Genetics (Ministry of Education), Department of Reproductive Endocrinology Women's Hospital, Zhejiang University School of Medicine Hangzhou China; ^3^ Shanghai Key Laboratory of Reproduction and Development Shanghai China; ^4^ Obstetrics and Gynecology Hospital, Institute of Reproduction and Development Fudan University Shanghai China; ^5^ Research Units of Embryo Original Diseases Chinese Academy of Medical Sciences (No. 2019RU056) Shanghai China; ^6^ Women's Hospital, School of Medicine Zhejiang University Hangzhou China

**Keywords:** *FBN1* gene, Marfan syndrome, novel variant, prenatal diagnosis

## Abstract

**Background:**

Marfan syndrome (MFS) is a complex genetic systemic connective tissue disorder. It is well known that genetic factors play a critical role in the progression of MFS, with nearly all cases attributed to variants in the *FBN1* gene.

**Methods:**

We investigated a Chinese family with MFS spanning two generations. Whole exome sequencing, in silico analysis, minigene constructs, transfection, RT‐PCR, and protein secondary structure analysis were used to analyze the genotype of the proband and his father.

**Results:**

The main clinical manifestations of the proband and his father were subluxation of the left lens and high myopia with pectus deformity. Whole exome sequencing identified a novel single nucleotide variant (SNV) in the *FBN1* gene at a non‐canonical splice site, c.443‐3C>G. This variant resulted in two abnormal mRNA transcripts, leading to a frameshift and an in‐frame insertion. Further in vitro experiments indicated that the c.443‐3C>G variant in *FBN1* was pathogenic and functionally harmful.

**Conclusion:**

This research identified a novel intronic pathogenic *FBN1*: c.443‐3C>G gene variant, which led to two different aberrant splicing effects. Further functional analysis expands the variant spectrum and provides a strong indication and sufficient basis for preimplantation genetic testing for monogenic disease (PGT‐M).

## Graphical abstract

We found a novel SNV in FBN1 at the non‐canonical splice site c.443‐3C>G in a Chinese family with Marfan syndrome (MFS) spanning two generations. This variant results in the formation of two abnormal mRNA transcripts, each leading to distinct aberrant splicing effects.

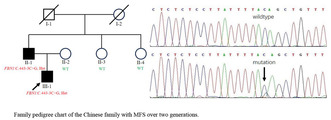

## 1 Introduction

Marfan syndrome (MFS) is a complex genetic systemic connective tissue disorder, first described in 1896. The estimated prevalence is 1 in 3000–5000 in Europe (Howarth et al. 2007; Moberg et al. 2012) whereas 1–2/10,000 in China (Dong et al. 2012), with no apparent sex or ethnicity biases. It is characterized by a wide range of clinical manifestations involving the skeletal, ocular, and cardiovascular systems, demonstrating striking pleiotropic effects and variable clinical symptoms. The diagnosis relies on defined clinical criteria (Ghent nosology), outlined by international experts to facilitate accurate recognition of this genetic aneurysm syndrome and to improve patient management and counseling (Loeys et al. 2010). However, the survival of MFS patients has been substantially improved through prophylactic aortic surgery combined with comprehensive medical treatment (Franken et al. 2015).

Fibrillin, encoded by the *FBN1* gene, is a major constitutive element of extracellular microfibrils and is widely distributed in both elastic and nonelastic connective tissue throughout the body, thereby providing stability and elasticity. The *FBN1* gene, located in 15q21.1 and comprising 65 exons, is the causative gene of a series of autosomal dominant connective tissue disorders. Moreover, pathogenic variations in *FBN1* account for the majority of MFS cases. It is evident that 1/4 to 1/3 of MFS cases are sporadic (Fleck et al. 2004), yet relevant clinical data provide evidence that MFS follows an autosomal dominant inheritance pattern (Du et al. 2021).

A Chinese family with MFS was investigated spanning two generations in this study. A novel SNV of *FBN1* at the noncanonical splice site c.443‐3C>G was found in the affected individuals. Further functional analysis classified the pathogenicity, pathogenesis, and classification of this variant, expanding the variant spectrum and providing a solid foundation for preimplantation genetic testing for monogenic disorders (PGT‐M).

## 2 Patients and Methods

### 2.1 Patients

This study was approved by the ethics committee of Women's Hospital, School of Medicine, Zhejiang University, and followed the tenets of the Declaration of Helsinki. The written informed consent was obtained from the members of the involved Chinese family. Residual peripheral venous blood samples after sequencing were used for further analysis.

### 2.2 Whole Exome Sequencing

WES was performed at GiantMed Diagnostics Laboratory using the IDT xGen Exome Research Panel v2.0 for variation screening. The genetic data analysis involved assessing hundreds of thousands of gene variants in conjunction with databases containing pathogenic variations, normal human genome data, a clinical characteristics database of 2000 known genetic diseases, and genetic data analysis algorithms.

**FIGURE 1:**
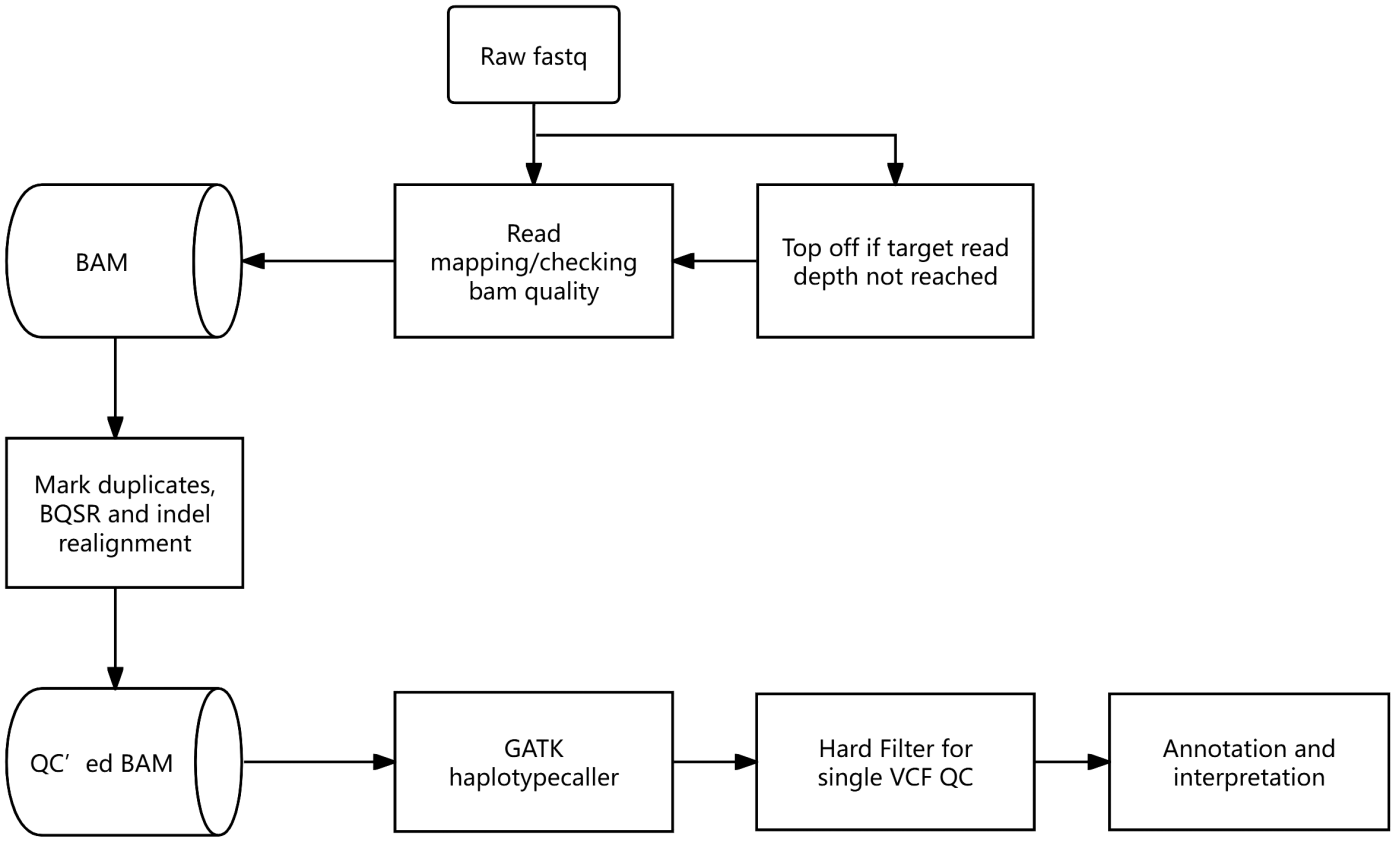
Variant detection algorithm of the “Whole exon sequencing”.

As shown in Figure 1, raw FASTQ files were analyzed using the in‐house DNA resequencing analysis workflow. First, sequencing reads were mapped to the hg19 reference genome using the BWA (v0.7.12‐r1039, Li and Durbin 2009) algorithm. The aligned SAM files were converted into BAM files and sorted using SAMtools (v0.1.18). Then PICARD (http://picard.sourceforge.net/, v1.134) was used to mark duplicate reads. GATK (v3.7, McKenna et al. 2010) was used to further improve BAM data quality by local read realignment near known insertion or deletion (indel) sites and base quality score recalibration (BQSR). Variants were called by Haplotype Caller (GATK v3.7, McKenna et al. 2010), while filter 1 was applied for SNVs and filter 2 for Indels. The sample was then annotated by ANNOVAR (2016 July 16 version). The Exome Aggregation Consortium (ExAC Version 0.3.1), 1000 Genomes Project, ESP6500, and other public databases were used to filter the variants.

### 2.3 DNA Extraction and Sanger Validation

Peripheral blood samples were collected from the proband and his parents, and DNA was extracted using the QIAamp DNA blood mini Kit (51104; QIAGEN). The sequencing primer was synthesized by Sangon Biotech (Shanghai) Co., Ltd., with its sequence included in the Supporting Information. DNA sequencing was performed by BGI Tech Solutions (Beijing Liuhe) Co., Ltd.

### 2.4 In Silico Analysis

The bioinformatic splicing tool SpliceAI (https://spliceailookup.broadinstitute.org/) was applied to predict the potential impact of variant on pre‐mRNA splicing using the donor or acceptor splice site signal with the default threshold. Primary structures were compared between the wild‐type and mutant sequences using ORF Finder (https://www.ncbi.nlm.nih.gov/orffinder/).

### 2.5 Minigene Construct, Transfection, and RT‐PCR

A pair of primers, including partial intron 5, exon 6, and partial intron 6, was designed to amplify genomic DNA from both variation carriers and normal controls, resulting in a product of 3027 bp in length. To form the final primer sequence, pSPL3 vector sequences containing the restriction endonucleases EcoR I and BamH I were added to the 5′ end of the primers (forward primer: 5′‐TTATGGGGTACGGGATCACCAGAATTCtgccctagtgaccatctgtg‐3′; reverse primer: 5′‐ACGGGATCACCAGATATCTGGGATCCtagggaccttcccaatgaca‐3′). The target fragment was subsequently cloned into the pSPL3 vector, and five monoclonal colonies were selected for each sample. Plasmid DNA was extracted and sequenced. The sequencing results were then compared with the standard sequence, and the correct plasmids were chosen for subsequent experiments.

The recombinant expression plasmid was then transfected into COS7 cells following the manufacturer's instructions using a PolyJet transfection reagent (SignaGen Laboratories, MD, USA). Following the extraction of total RNA from the cells and reverse transcription, specific primers (F: TCTGAGTCACCTGGACAACC; R: ATCTCAGTGGTATTTGTGAGC) were used to amplify the resultant cDNA. The PCR products were sequenced after fractionation using 3% agarose gel electrophoresis.

### 2.6 Protein Secondary Structure Analysis

Protein structure homology modeling and protein structural effect evaluation were analyzed utilizing Alphaflod Colab. The Uniprot ID P35555 was entered to obtain the reference sequence and the residues and variants were selected to finalize the analysis.

### 2.7 The pSPL3‐Based Minigene Splicing Assay

To study and determine the function of the mutation at the transcriptional level, an exon trapping study was conducted. DNA from the proband, harboring one wild type (WT) and one mutant allele, was used as a template to generate a genomic fragment containing intron 5, exon 6, and partial intron 6. This fragment was cloned into the pSPL3 exon trapping vector through double digestion by BamHI and XhoI. WT and mutant plasmids were transiently transfected into African green monkey kidney fibroblast‐like cell line (COS‐7) using Superfect reagents (Westburg, Leusden, The Netherlands). COS‐7 cells were cultured in Dulbecco's modified Eagle's medium supplemented with 10% fetal bovine serum, 1% penicillin–streptomycin, and 1% glutamine in a humidified, 5% CO_2_ incubator at 37°C. At 48‐h post‐transfection, total RNA was extracted with Trizol. cDNA was prepared using 5 μg total RNA in a total volume of 20 μL with Superscript II RNAse H‐reverse transcriptase and oligo‐dT priming (Invitrogen). Amplification products obtained by PCR with vector primers SD6 and SA2 were separated on a 2% TAE agarose gel and characterized by direct sequencing. The RT‐PCR products (cDNA) were subjected to fragment analysis on 3730 DNA Analyzer (Applied Biosystems, Foster City, CA). The ratios of the different transcripts were calculated based on the peak heights of individual fragments.

## 3 Results

### 3.1 Clinical Evaluation

**FIGURE 2:**
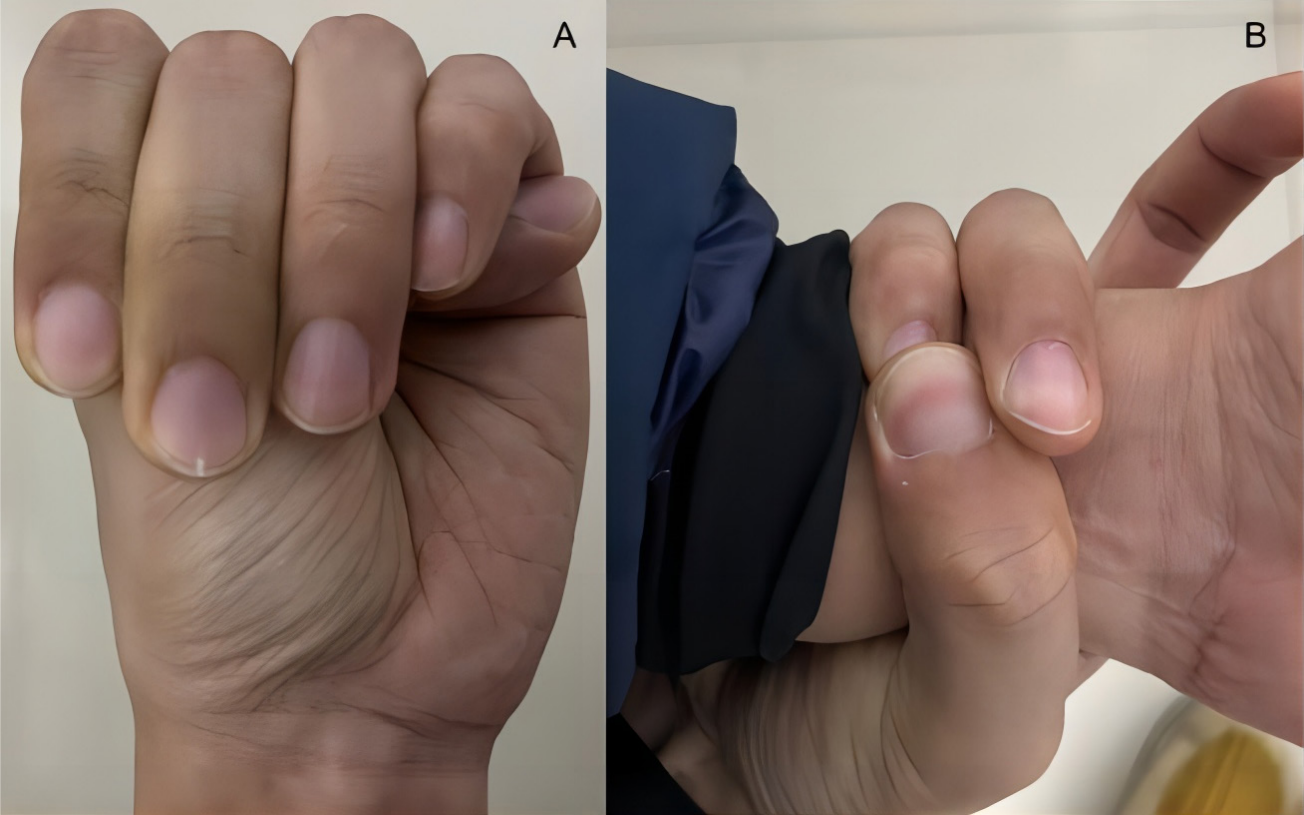
Clinical features (A) showed thumb sign (distal phalanx of the thumb beyond the edge of a clenched fist) and (B) reflected abnormally long fingers.

The main clinical manifestations of the proband were subluxation of the left lens, high myopia, pectus, and arachnodactyly (Figure 2). His father had similar symptoms. Based on the revised Ghent nosology for Marfan syndrome, the systemic feature score of the participants in the current research was 7 (Table 1), indicating a diagnosis of Marfan syndrome.

**TABLE 1:** Scoring of systemic features.

	Proband	Score
Wrist and thumb sign	+	3
Pectus carinatum deformity	+	2
Hindfoot deformity		
Pneumothorax		
Dural ectasia		
Protrusio acetabuli		
Reduced US/LS and increased arm/height and no severe scoliosis		
Scoliosis or thoracolumbar kyphosis		
Reduced elbow extension		
Facial features	+	1
Skin striae		
Myopia	+	1
Mitral valve prolapse (all types)		
Total		7

*Note:* Maximum total: 20 points; score ≥7 indicates systemic involvement; US/LS, upper segment/lower segment ratio. Face features include dolichocephaly, enophthalmos, downslanting palpebral fissures, malar hypoplasia, retrognathia. Maximum total: 20 points—the scoring system has a maximum possible score of 20 points. Score ≥7 indicates systemic involvement. If the score is 7 or higher, it suggests that there is involvement of multiple systems in the body, indicating a broader or more severe condition. US/LS Ratio: The ratio of the upper segment to the lower segment of the body; facial features include dolichocephaly, enophthalmos, down slanting palpebral fissures, malar hypoplasia, retrognathia.

### 3.2 Variant Detection and Validation

**FIGURE 3:**
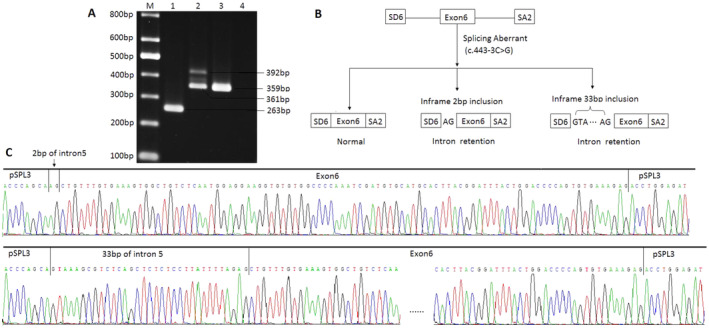
Minigene results of FBN1:c.443‐3C>G. (A) M, Mark; Lane 1, empty vector result; Lane 2, mutant result showed long/short band; Lane 3, wild‐type result; Lane 4, blank control. (B) Splicing models schematic representation. (C) Sanger sequencing results of minigene analysis.

Whole exome sequencing revealed that the proband and his father had a heterozygous variant in *FBN1* (c.443‐3C>G, ENSG00000166147). This was confirmed by Sanger sequencing. The aforementioned variant was not recorded in any of the reference databases, such as gnomAD, ClinVar, and HGMD. The online software SpliceAI predicted an Acceptor Loss score of 0.95, which reflects the potential aberrant splicing. The results of in vitro experiments indicated that the wild‐type plasmid produced the expected 263‐bp transcript, while two abnormal mRNA bands of 361 bp and 392 bp were detected in the mutant plasmid by Sanger sequencing. This indicated that a shorter segment resulted from the inclusion of the two nucleotides, AG, at the end of the 3′ splice site of intron 5, forming a premature termination codon. While the other led to a 33 bp in‐frame insertion (Figure 3).

### 3.3 Protein Structure Effect Evaluation

**FIGURE 4:**
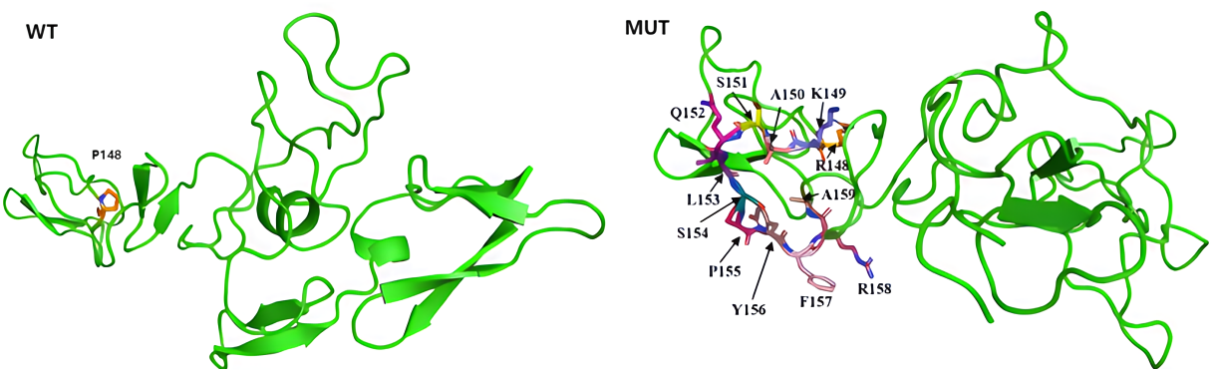
3D structure prediction of FBN1. The 33 bp in‐frame insertion led to Pro148 residue is replaced with Arg‐Lys‐Ala‐Ser‐Gln‐Leu‐Ser‐Pro‐Tyr‐Phe‐Arg‐Ala residues, which resulting in an increase in protein length and a change in protein folding, suggesting that the mutation may affect the 3D structure of the protein.

In the structure homology modeling (Figure 4), it is evident that the chain is extended. The Pro148 residue is replaced with Arg‐Lys‐Ala‐Ser‐Gln‐Leu‐Ser‐Pro‐Tyr‐Phe‐Arg‐Ala residues as a result of the 33 bp in‐frame insertion. This alteration may disturb the domain and affect its function by introducing amino acids with different properties.

### 3.4 Classification

These observations indicate that the variant c.443‐3C>G in *FBN1* is functionally harmful and disease‐causing. According to the ACMG guideline (2015), it should be classified as likely pathogenic.

## 4 Discussion

It is well known that Marfan syndrome (MFS) was first reported in 1896, and the fibrillin‐1 (*FBN1*) gene was first isolated from cDNA clones in 1991 (Dietz et al. 1991). According to recent studies, genetic factors play a critical role in the progression of MFS (Verstraeten et al. 2016). About 75% of the affected individuals with the classic phenotype have variants over generations, while the remaining 25% have de novo variants (Franken et al. 2015). Up to now, more than 3000 variants of *FBN1* in individuals with MFS have been reported, and the proportion of point variants and large rearrangements are 73.09% and 1.66%, respectively (Becerra‐Munoz et al. 2018; Yang et al. 2018). Variants are distributed throughout the entire gene and there is no obvious aggregation or periodicity. Even though it is widely believed that MFS follows an autosomal dominant pattern, it is uncommon that MFS influenced with biallelic variations in FBN1 is associated with severe clinical manifestations (Li et al. 2019).

In this case, only a non‐class splice site *FBN1*: c.443‐3C>G was detected, while minigene analysis was used to investigate the mechanism of variation‐induced alternative splicing. The results showed that it forms two abnormal mRNA transcripts, one including a frameshift and the other an in‐frame insertion.

Previous studies have identified cryptic splicing in Marfan syndrome, involving a 111‐bp insertion and a 48‐bp deletion (Karttunen et al. 1998), both of which caused premature stop codons in *FBN1*, resulting in shorter *FBN1* polypeptides. Another case described a 33‐bp insertion encoding 11 amino acids in fibrillin‐1, including two cysteines (Hutchinson et al. 2001). Splice site variants in MFS have typically been associated with deletions or exon skipping (Li et al. 2020; Liu et al. 1997). The majority of exon‐skipping cases are in‐frame, resulting in incomplete proteins lacking integral domain. In a small percentage of cases, exon skipping causes frameshift and downstream premature termination codons (PTCs), which can initiate nonsense‐mediated mRNA decay (NMD). In this case, one of the aberrant mRNA transcripts includes the insertion of two nucleotides (AG) at the end of the 3′ splice site, which may be relevant to the HI (Haploinsufficiency) mechanism. The other transcript involves an in‐frame insertion resulting from a splice site variant. A previous study demonstrated that an intronic variant abolished the canonical splicing site, leading to the activation of two cryptic splicing sites, resulting in an insertion (Anna and Monika 2018). In contrast, both transcripts in this study formed PTCs and activated the NMD mechanism.

The above findings can be considered evidence for PS3, a criteria for classifying pathogenic variants according to the ACMG guideline (2015): well established in vitro or in vivo functional studies supportive of a damaging effect on the gene or gene product. It is noted that functional studies validated, reproducible, and robust in a clinical diagnostic laboratory setting are considered the most well established. Therefore, combined with two additional lines of evidence: PM2_strong (absent from controls or presence at extremely low frequency in the Exome Sequencing Project, 1000 Genomes Project, or Exome Aggregation Consortium) and PP4 (the patient's phenotype or family history is highly specific for a disease with a single genetic etiology), this variant should be classified as “Likely pathogenic.”

By far, over 3000 variants of *FBN1* in MFS patients have been reported, with point variants and large rearrangements comprising 73.09% and 1.66% of cases, respectively (Becerra‐Munoz et al. 2018; Yang et al. 2018). These variants are distributed throughout the entire gene, with no apparent aggregation or periodicity. While MFS is widely recognized as following an autosomal dominant pattern, rare cases of MFS with compound heterozygous variants in *FBN1* have been reported, and biallelic variants in *FBN1* are associated with severe clinical manifestations (Li et al. 2019).

In a typical autosomal dominant disease such as Marfan syndrome (MFS), the pathogenic mechanisms primarily involve dominant‐negative (DN) effect and haploinsufficiency (HI), depending largely on the variant types (Franken et al. 2015). Previous research has established criteria for classifying different variant types. Variants leading to exon skipping or deletions resulting in in‐frame events and consequently producing shorter, stable proteins are classified as DN cases (Liu et al. 2001). In contrast, deletions of the entire *FBN1* gene or exon events that impact transcription and/or translation, along with variants leading to very short truncated proteins, are classified into the HI group (Franken et al. 2015). Missense variants that result in stable mutant fibrillin protein with altered structure or degradation are classified based on their effects on protein stability and structure (Schrijver et al. 1999). PTCs or frameshifts are categorized based on their potential to trigger nonsense‐mediated decay (NMD) (Schrijver et al. 2002). Although this theoretical classification appears reasonable, experimental evidence has demonstrated that predicting the ultimate effect of an *FBN1* variant on the protein is not straightforward. Therefore, without experimental protein studies, the predicted effects of a particular variant remain speculative. There has been long‐standing debate about whether *FBN1* variants cause Marfan syndrome through HI, DN mechanisms, or both (Dietz 2015). DN variants of *FBN1* affect the stability of microfibers under various conditions, rather than microfibril assembly, leading to different pathological manifestations (Charbonneau et al. 2010). As a result, HI mutations are theoretically expected to produce a consistent phenotype (Landis et al. 2017). Without considering the variant classification, there is a well‐established correlation between genotype and phenotype, depending on the variant's position within specific codons and domains. The most commonly accepted variants are located in exons 24–32, which are associated with early‐onset, neonatal issues, and more severe phenotypes (Faivre et al. 2007; Peng et al. 2016; Schrijver et al. 2002). Patients with variants in exons 43–65 exhibit an increased frequency of major cardiovascular events (Arbustini et al. 2005; Chung et al. 2009; Gao et al. 2019). Additionally, variants that alter the structure of *FBN1* are associated with symptoms in various affected organs or tissues (Faivre et al. 2007; Reinhardt et al. 2000). In our case, both pathogenic mechanisms might be simultaneously activated, suggesting the patient should exhibit more severe symptoms. However, the observed clinical manifestations were inconsistent with this prediction, indicating that a new hypothesis may be needed to explain this phenomenon.

One limitation of this study is that the construct utilized incorporated only a portion of intron 5, the complete sequence of exon 6, and a section of intron 6. Consequently, defects in the splicing process affecting the exon 5/exon 6 and exon 6/exon 7 junctions would remain undetected. This limitation is significant because it means that one of the most common consequences of variations in the *FBN1* gene, specifically the skipping of exon 6 during splicing, would not be detected with this particular construct. Therefore, while the construct's design is useful for studying other aspects of *FBN1* gene expression and splicing, it is not suitable for identifying splicing defects at the exon 5/6 and 6/7 junctions.

When studying splicing defects in probands with genetic disorders, considering their unique genetic backgrounds is crucial. In the case of the *FBN1* gene associated with Marfan syndrome and other related connective tissue disorders, mutations affecting splicing can have significant clinical implications. For example, as previously noted, the most common result of *FBN1* splice mutations is a skip in exon 6. However, other splicing alterations are possible, and the proband's genetic background can influence the risk and nature of these defects. Various factors, such as genetic variation, epigenetic markings, and expression levels of splicing factors, add complexity to the situation. However, genetic analysis and identification of the upper lineage were not carried out, representing another limitation of this study, as the influence of genetic background on the splicing defect could not be fully understood.

This study utilized non‐human primate cells, providing insights into splicing mechanisms and gene function; however, extrapolating these findings to humans is limited by genetic differences and variations in cell environments. Although validation using human cells or tissues would be ideal, it was not feasible in this study.

## 5 Conclusion

This research identified a novel intronic pathogenic *FBN1* variant (c.443‐3C>G) in a family with MFS, leading to two distinct aberrant splicing effects. These findings thus expanded the variant spectrum and provided a strong indication and sufficient basis for PGT‐M.

## Author Contributions

**Djouhayna Dougarem:** conceptualization (leading), investigation (equal), writing – original draft (equal), review and editing (equal). **Yi‐Xiao Chen:** methodology (leading), investigation (equal), data curation (equal), writing – original draft (equal), review and editing (equal). **Yi‐Na Sun:** review and editing (equal), investigation (equal), formal analysis (leading). **He‐Feng Huang:** funding acquisition, supervision. **Qiong Luo:** supervision, investigation.

## Conflicts of Interest

The authors declare no conflicts of interest.

## Supporting information


Supporting Information S1.

## Data Availability

The data that support the findings of this study are available on request from the corresponding author. The data are not publicly available due to privacy or ethical restrictions.
