# Supplementary material for: A Novel Heterozygous Intronic FBN1 Variant Contributes to Aberrant RNA Splicing in Marfan Syndrome
Source: Mol Genet Genomic Med. 2024 Sep 2;12(9):e70004. doi: 10.1002/mgg3.70004 (PMC11366968; doi:10.1002/mgg3.70004)
Supplement: Supplementary file 1 — Supporting Information S1. [file MGG3-12-e70004-s001.docx]

| FBN1-6F | 5' ACATTTTATTGTTGTCCTTCCAGAG 3' |
| --- | --- |
| FBN1-6R | 5' AGCTCAGCAACATTCAGGAAGTAG 3' |
| TRAPPC2-5F | 5' ATTGGCTGTTTCTGTTGAGATGTAG 3' |
| TRAPPC2-5R | 5' TACTAAGAAGGTAAGGATATGCCCC 3' |
